# Questioning the role of amygdala and insula in an attentional capture by emotional stimuli task

**DOI:** 10.1002/hbm.25290

**Published:** 2020-11-20

**Authors:** Michael Marxen, Mark J. Jacob, Lydia Hellrung, Philipp Riedel, Michael N. Smolka

**Affiliations:** ^1^ Department of Psychiatry and Neuroimaging Center Technische Universität Dresden Dresden Germany; ^2^ Department of Economics University of Zurich Zurich Switzerland

**Keywords:** amygdala, attentional capture, emotions, fMRI, insula, neuroimaging

## Abstract

Our senses are constantly monitoring the environment for emotionally salient stimuli that are potentially relevant for survival. Because of our limited cognitive resources, emotionally salient distractors prolong reaction times (RTs) as compared to neutral distractors. In addition, many studies have reported fMRI blood oxygen level‐dependent (BOLD) activation of both the amygdala and the anterior insula for similar valence contrasts. However, a direct correlation of trail‐by‐trial BOLD activity with RTs has not been shown, yet, which would be a crucial piece of evidence to relate the two observations. To investigate the role of the above two regions in the context of emotional distractor effects, we study here the correlation between BOLD activity and RTs for a simple attentional capture by emotional stimuli (ACES) choice reaction time task using a general linear subject‐level model with a parametric RT regressor. We found significant regression coefficients in the anterior insula, supplementary motor cortex, medial precentral regions, sensory‐motor areas and others, but not in the amygdala, despite activation of both insula and amygdala in the traditional valence contrast across trials (i.e., negative vs. neutral pictures). In addition, we found that subjects that exhibit a stronger RT distractor effect across trials also show a stronger BOLD valence contrast in the right anterior insula but not in the amygdala. Our results indicate that the current neuroimaging‐based evidence for the involvement of the amygdala in RT slowing is limited. We advocate that models of emotional capture should incorporate both the amygdala and the anterior insula as separate entities.

## Graphical abstract

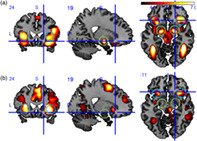

## SIGNIFICANCE STATEMENT

A core aspect of evolutionary survival is the ability to detect potential dangers and procreation opportunities. How the brain processes such emotionally salient distractors is a long‐standing debate within cognitive neuroscience and addressed by many neuroimaging studies. While the amygdala is often regarded as a pivotal region in this context, our study emphasizes the role of the insula based on the observation that insula but not amygdala activity is correlated with reaction times in an attentional capture by emotional stimuli task. Lack of methodological sensitivity and stimulus selection are shown to be severe limitations for the interpretation of neuroimaging data.

## 1 INTRODUCTION

During evolution, it has been an important aspect of brain development that we need to evaluate stimuli in our vicinity even when they are not related to our current activities. We need, for example, to detect potential threats or mates. For this purpose, we are constantly monitoring background stimuli and evaluate them emotionally. If a stimulus is regarded as important, it captures our attention and cognitive resources are allocated for further evaluation.

Experimentally, this leads to the phenomenon that emotionally charged stimuli are more effective distractors than neutral stimuli as measured by reaction times (RTs), error rates, or working memory performance. This emotional capture effect is also reflected in measures of neural activity such as event‐related potentials (ERPs) and blood oxygen level‐dependent functional magnetic resonance imaging (BOLD fMRI). Brain regions facilitating the perception of emotions include the amygdala, the anterior insula, the orbital frontal cortex, and other areas (Carretie, 2014; Carretie, Albert, Lopez‐Martin, & Tapia, 2009; Costafreda, Brammer, David, & Fu, 2008; Dolcos, Iordan, & Dolcos, 2011; Iordan, Dolcos, & Dolcos, 2013; Lindquist, Wager, Kober, Bliss‐Moreau, & Barrett, 2012; Mitchell et al., 2008; Phan, Wager, Taylor, & Liberzon, 2002; Scharpf, Wendt, Lotze, & Hamm, 2010). The common cognitive model in this context is that the amygdala acts as an early relevance detector, which influences the distribution of available processing resources in a bottom‐up (stimulus‐driven) way. Negative images are regarded as more relevant than neutral ones and, thus, are drawing more resources away from the task at hand.

A number of reviews highlight the importance of the amygdala for the processing of emotional stimuli (Armony, 2013; Iordan et al., 2013; LeDoux, 2003; Pessoa, 2010, 2017; Sander, Grafman, & Zalla, 2003; Sergerie, Chochol, & Armony, 2008; Zald, 2003). Functionally and structurally, the amygdala is connected with the prefrontal cortex (M. J. Kim et al., 2011; Riedel et al., 2019), and has been described as an integral part of the” hot emotional system” with inputs from the temporal, frontal and insular cortices (Dolcos et al., 2011; Iordan et al., 2013; Pessoa, 2017). Clinically, group differences in amygdala activity are, for example, observed for subjects with or without a family history of depression (Pilhatsch et al., 2014) or in subjects with major depressive disorder (Davidson, Pizzagalli, Nitschke, & Putnam, 2002; Sheline et al., 2001). Furthermore, anxious or phobic subjects show a stronger activation of the amygdala (Bishop, Aguirre, Nunez‐Elizalde, & Toker, 2015; Straube, Mentzel, & Miltner, 2006).

The anterior insula frequently co‐activates with the amygdala when studying effects of valence (Dolcos & McCarthy, 2006; Lim, Padmala, & Pessoa, 2008; Lindquist, Satpute, Wager, Weber, & Barrett, 2016; Pessoa, McKenna, Gutierrez, & Ungerleider, 2002; Pessoa, Padmala, & Morland, 2005; Scharpf et al., 2010). While the insula is not explicitly regarded a part of the above hot emotional system, it is known to be involved in numerous brain functions such as somatosensation, sensorimotor activity, hearing, pain perception, conflict monitoring, task switching, inhibition, etc. and also in the processing of emotions (Chang, Yarkoni, Khaw, & Sanfey, 2013). Lindquist et al. (Lindquist et al., 2016) found in a meta‐analysis of close to 400 imaging studies that both the amygdala and the insula are valence general brain regions, meaning activate for both negative versus neutral and positive versus neutral imaging contrasts. Both regions, in concert with the dorsal anterior cingulate cortex (dACC), the pre‐supplementary motor area (preSMA) and other structures, are often considered part of a brain network called the “salience network” that is correlated with arousal, as, for example, measured by heart rate. The salience network is thought to engage the executive control network, composed primarily of ventral‐ and dorsolateral prefrontal cortex (v/dlPFC) and lateral parietal cortex, when required (Seeley et al., 2007; Young et al., 2017). Actually, studies of salience network function sometimes have a particular focus on the anterior insula. Some authors, for example, are assigning the anterior insula a causal role in switching between executive control and default mode brain networks (Menon & Uddin, 2010; Sridharan, Levitin, & Menon, 2008). Importantly, the subregions of the salience network do not always seem to act in concert. For example, Kim et al. (J. Kim et al., 2020) found that the anterior insula, among other brain regions, was predictive of arousal ratings during movie watching, while the amygdala was not. In the context of attentional capture and early processing (preattention/evaluation) of emotional stimuli, the specific roles of anterior insula and amygdala are still unclear (Carretie, 2014) and addressed in our study.

Implicitly, we tend to take the coincidence of slower RTs with higher brain activity for negative distractor images, for example in the amygdala, as evidence that the signal in this region is responsible for the RT slowing. However, to study how brain regions influence human behavior requires ideally an experiment that produces both behavioral as well as brain imaging effects and that allows us to correlate one with the other on a trial‐by‐trial level. Surprisingly, the review of Carretie (Carretie, 2014) of 55 “concurrent but distinct target‐distractor paradigms” includes only 5 studies that reported both fMRI and behavioral effects of stimulus valence and not a single study that reported a direct trial‐by‐trial correlation between behavior and the imaging activity that could demonstrate a shared source of variance. In this paper, we will present such data for a simple attentional capture by emotional stimuli (ACES) choice RT task.

We will investigate the neural underpinning of the “attention capture by emotional distractors” effect by initially considering behavioral RT slowing and the conventional categorical fMRI contrast between negative and neutral distractors. Furthermore, we will identify brain regions with BOLD signals that correlate with RTs using a parametric fMRI model within subjects. If our measurements are sensitive enough, we would expect that regions crucial for the RT slowing should show activation for both contrasts. We will especially focus on the anterior insula and the amygdala. Additionally, we will investigate in our subject group whether the RT capture effect between subjects is correlated with the BOLD contrast between negative and neutral distractor images in these two regions. Observing a correlation would provide further evidence for the involvement of these regions in the capturing process. Important limitations of this approach will be discussed in detail.

## 2 MATERIALS AND METHODS

### 2.1 Participants

Forthy‐nine healthy, right‐handed (Edinburgh Handedness Inventory [Oldfield, 1971] score > = 50) adults between the ages of 18 to 40 years performed the ACES experiment. Of these, 44 subjects (mean age = 24.5, std = 4.1, 20 male) could be included in the behavioral analysis, five were excluded because of more than 10 missed or incorrect responses in 120 trials. After quality control of the imaging data, four more subjects were excluded due to translational motions within the run larger than 3 mm, leaving 40 subjects (mean age = 24.2, std = 3.6, 18 male) for the fMRI analysis of the ACES task.

Exclusion criteria at recruitment included a history of mental disorder, physical conditions that prevent lying comfortably inside an MRI scanner, a body mass larger than 120 kg, vision impairments outside of −5 – +3 diopters, insufficient knowledge of the German language, potential pregnancy, and contra‐indications for MR‐scanning. The study was approved by the Ethics Review Board of the Technische Universität Dresden. All subjects signed informed consent forms after receiving a detailed description of the experiment. Most subjects also took part in a subsequent fMRI neurofeedback study (Marxen et al., 2016) and received approximately €90.00 compensation for participating in this study on the last experimental day.

### 2.2 Experimental design

The reported task was administered on the first experimental day of a larger study. Details of the recruitment, screening, and objectives of this study can be found in Marxen et al. (2016). On Experimental Day 1, a T1‐weighted MRI, a resting state fMRI sequence, the ACES task, and a further task (Riedel et al., 2016) were acquired. We will focus here on the ACES task (see below for details).

### 2.3 The ACES task

**FIGURE 1:**
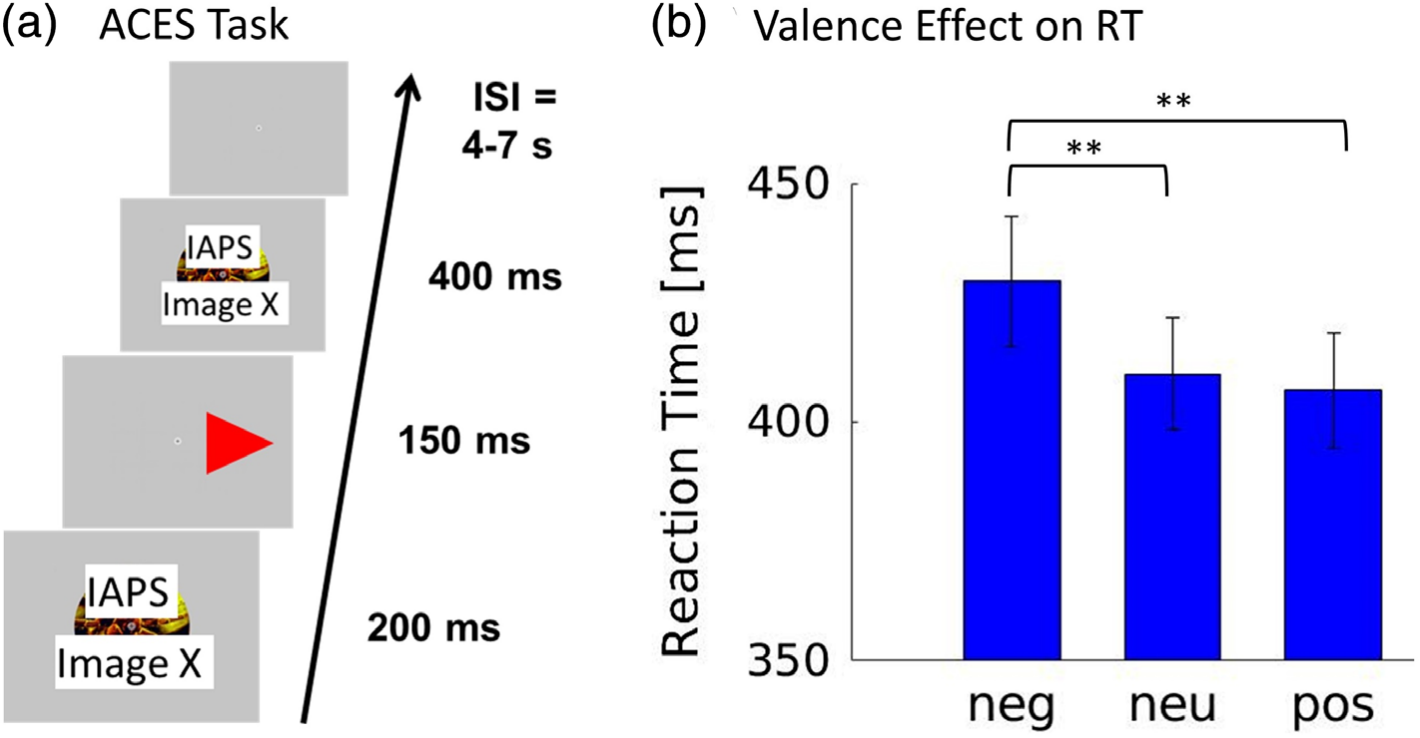
(a) Illustration of the attentional capture by emotional stimuli (ACES) choice reaction time (RT) task. (b) Effect of image valence category (neg – negative, neu – neutral, pos ‐ positive) on mean RT with standard errors in *N* = 44 subjects: **p < .0005. There was no significant difference in RTs between neutral and positive distractors

In the ACES task, a choice RT task is performed in between the display of emotional images (same image before RT cue and after). The images have a variable emotional valence. The ACES task operationalizes valence/arousal effects (or “attentional capture”) as response slowing. The task was adapted from (Mitchell et al., 2008) and is illustrated in Figure 1(a). Our primary adaptation was that we replaced the reaction cues (a circle and a square) used by Mitchell et al. by a red triangle pointing right positioned to the right of a central fixation cross and a left pointing triangle positioned to the left of the fixation cross. Subjects were asked to press “the left” button with their right index finger as fast as possible for the left pointing triangle and “the right” button with their right middle finger for the right pointing triangle. We introduced this alteration because pilot studies indicated a larger emotional distractor effect on RTs for more intuitive tasks (with less cognitive load). This is in line with the attentional blindness literature (Mack, 1998; Simons, 2000; Simons & Ambinder, 2005), which indicates that cognitive load increases attentional blindness. In addition, a higher cognitive load is known to lead to a lower activity of the amygdala and reduced perception of emotional stimuli (Bishop, Jenkins, & Lawrence, 2007; Lim et al., 2008; Pessoa et al., 2005; Silvert et al., 2007; Van Dillen, Heslenfeld, & Koole, 2009), and the review by Carretie et al. (Carretie, 2014) indicates that perceptual tasks show stronger behavioral effects than nonperceptual, presumably more complex tasks.

The timing of a trial was the same as in (Mitchell et al., 2008): 200 ms for the initial image display, 150 ms for the presentation of the task cue, and 400 ms for the final image display. An advantage of temporal flankers is that no saccades are required to capture the emotional content (McSorley, Cruickshank, & Inman, 2009). The duration of the initial display of 200 ms is reasonable to allow detection of emotional valence, for example, based on findings that confident recognition of emotional facial expression is possible beyond a display time of 100‐150 ms in a backward masking experiment (Esteves & Ohman, 1993). Distractor images were mostly taken from the International Affective Picture System (IAPS) (Lang, Bradley, & Cuthbert, 2005) and modified such that the primary emotional stimulus was cut out circularly and centered on the screen. A fixation cross was also added at the center of the screen. This was done to avoid additional response slowing related to visual search phenomena. The circular image occupied a visual angle of 9° for the subject. Images and triangles were presented on a gray background. The experiment was programmed in Presentation® software (Version 16.3, Neurobehavioral Systems Inc., Berkeley, CA).

Negative, neutral, and positive images (40 for each category) were presented as distractors. Images for female/male subjects were assigned to valence categories according to female/male IAPS ratings. The average valence rating (mean ± std) for females was 1.9 ± 0.7 / 5.6 ± 0.7 / 7.5 ± 0.5 and for males 2.6 ± 0.7 / 5.4 ± 0.6 / 7.0 ± 0.4 for negative / neutral / positive images, respectively. A table with the used images and valence/arousal ratings is part of the Supplement (Table S4). During the inter‐simulus‐intervals (ISI) between trials of 4, 5, 6, or 7 s (mean 5.5 s), a fixation cross was shown. Three different temporal orders of distractor images and ISIs were employed.

### 2.4 fMRI data acquisition

Images were acquired on a 3‐Tesla Siemens Tim Trio scanner using the Siemens 32‐channel head coil (Siemens, Erlangen, Germany). T1‐weighted images were acquired with a 3D magnetization‐prepared rapid gradient echo (MP‐RAGE) sequence (repetition time (TR) = 1.9 s, echo time (TE) = 2.26 s, field of view (FOV) = 256 x 224 x 176 mm^3^, voxel size = 1 x 1 x 1 mm^3^, inversion time = 0.9 s, flip angle (FA) = 9°, phase partial Fourier 7/8, bandwidth (BW) = 200 Hz/Px). To maximize BOLD sensitivity in the amygdala, which suffers considerable susceptibility‐related signal losses, we employed a multi‐echo EPI sequence and fixed TE‐dependent echo weights {0.59, 0.90, 1, 0.97, 0.88, 0.77}, which were selected for an average T_2_^*^‐value of 30 ms in the amygdala (Posse et al., 1999; Posse et al., 2003). Functional data were acquired with 6 echoes (TR = 2.54 s, TE = {8.6, 18.3, 28, 38, 48, 57} ms, FOV = 192 x 192 x 132 mm^3^, voxel size = 4 x 4 x 3.2 mm^3^ with a slice gap of 25%, GRAPPA with ipat factor 3 and 42 reference lines, FA = 82°, BW = 2084 Hz/Px, slice orientation A > C, slice order: descending). The parallel imaging acceleration was chosen to minimize geometrical distortion in the amygdala due to susceptibility inhomogeneity without incurring significant increase in parallel imaging‐related noise. EPI images were distortion‐corrected in real‐time based on point spread function mapping using a phase‐encoded prescan with a single TE = 8.7 ms (Zaitsev, Hennig, & Speck, 2004).

### 2.5 Analysis of reaction times

The behavioral data was analyzed using SPSS Statistics 24 (IBM SPSS Statistics, Armonk, New York) and MATLAB R2017b (The Mathworks, Inc., Natick, MA). As the first two trials were found to be outliers (a value that is more than three scaled median absolute deviations away from the median) in many subjects (29 for the first trial and 9 for the second of 44), they were excluded from the analysis. A trial was considered as “incorrect” and not included for further analyses if the directional response was wrong or no response was recorded within 1,600 ms of cue onset. To test for an effect of distractor valence on RTs, a repeated‐measures ANOVA was performed with the within‐subject factor valence with three levels (negative [neg], positive [pos], neutral [neu]). Subsequent one‐sided paired t‐tests were conducted for mean(RT_neg_) > mean(RT_neu_), mean(RT_neg_) > mean(RT_pos_) and mean(RT_pos_) > mean(RT_neu_). Uncorrected P‐values will be given. Group mean RT‐values will be given with standard errors. As a measure of the distractor effect size, we will quote Cohen's d = (mean(RT_neg_)‐mean(RT_neu_))/sqrt(mean(var(RT_neg_), var(RT_neu_)) and d_z_ = mean(ΔRT_neg‐neu_)/std(ΔRT_neg‐neu_).

### 2.6 Analysis of fMRI data

The fMRI data were preprocessed using SPM8 (Wellcome Department of Cognitive Neurology, London, UK) including slice‐time correction, realignment (motion correction), T1‐based normalization with 2 mm isotropic voxels, and smoothing with an 8 mm FWHM kernel. Note that distortion correction was done in real‐time previously (see Section 2.4). Statistics are computed using SPM12. Table 1 provides a summary of the relevant research questions of this paper and the associated models. Models 1–3 investigate fMRI signals as the dependent variable; Model 4 treats ΔRT as the dependent variable.

**TABLE 1:** Summary of computed models, the addressed question and the obtained answers

Model #	Question	Dep. variable	Model name	Contrast of interest	Result
1	Is Amy/Ins BOLD activity larger for negative distractors than for neutral ones?	BOLD	Valence model	Negative > neutral (subject‐level)	Yes, the BOLD signal is larger for negative distractors in both Amy and Ins.
2	Is Amy/Ins BOLD activity correlated with RT within subjects?	BOLD	RT model	Correlation with RT > 0 (subject‐level)	Only Ins signal is correlated with RT.
3	Is the negative > neutral contrast in the Amy/Ins correlated with the associate valence effect on RT between subjects?	BOLD	ΔRT covariate model	Correlation of negative > neutral contrast with ΔRT [mean(RT_neg_) – Mean(RT_neu_)]	The valence BOLD contrast is correlated with RT contrast only in the right Ins.
4	Is the valence effect on RT between subjects correlated with the negative > neutral contrast in the Amy/Ins?	ΔRT	ΔRT versus BOLD model	GLM coefficients for negative > neutral BOLD contrasts within Amy/Ins	Only the Ins valence BOLD signal contrast explains the RT contrast.

We computed two different subject‐level GLM models with events at the initial image onset: a) we generated three valence regressors (“negative”, “positive”, “neutral”) for the “Valence Model” (Table 1: Model #1; also see below) to investigate the effect of distractor valence on BOLD activity. b) we used a main onset regressor for all correct trials and a mean‐free parametric RT regressor for the “RT Model” (Table 1: Model #2; also see below) to investigate brain activity correlated with RT on the subject level. Both models contained additional single event regressors for missed or incorrect trials. All of the above regressors were convolved with SPM's canonical hemodynamic response function. Six motion regressors were also included.

On the group level, three models (Table 1: Models #1–3) were estimated: 1. In the “Valence Model” (Model #1), a full factorial model with the factor valence and the above three levels was used to investigate whether BOLD activity in the target regions is larger for negative trials than for neutral ones. 2. In the “RT Model” (Model #2), regression coefficients of the parametric RT regressor were taken to the group level for a random effects analysis using a one‐sample *t*‐test to test whether BOLD activity is correlated with RT within subjects. 3. The “ΔRT Covariate Model” (Model #3) investigated the significance of the RT valence distractor effect “ΔRT = mean(RT_neg_) ‐ mean(RT_neu_)” in each subject as a covariate in a one‐sample t‐test of the “Negative > Neutral” contrast from the first level “Valence Model”. The hypothesis that a stronger RT distractor effect is reflected by a stronger fMRI “Negative > Neutral” contrast between subjects would be reflected through a positive covariate coefficient.

On the group level, a cluster threshold of *p* < 0.001 and family‐wise error correction (FWE) for a small volume (SVC) was used with a mask (region‐of‐interest [ROI] with 2,636 2 mm‐isotropic voxel) that contained the bilateral anterior insula (based on the Neuromorphometrics Atlas contained in SPM12^1^) and the bilateral amygdala (based on the SPM Anatomy Toolbox Version 1.8[Amunts et al., 2005; Eickhoff et al., 2005]). For this analysis, no minimum cluster size was used. The SVC approach was chosen to reduce the risk of false negative findings. Whole‐brain FWE‐corrected results with a minimum cluster size of 50 voxels will also be given in the Supplement.

Lastly in the “ΔRT versus BOLD Model” (Table 1: Model #4), we investigated the RT distractor effect ΔRT as a dependent variable of both the BOLD contrasts in the bilateral amygdala and in the bilateral anterior insula. To this end, we extracted the mean “Negative‐Neutral” contrast from both areas using the above masks and fitted a multiple linear regression model with these two regressors and a constant. This addresses the question whether a strong fMRI effect in a particular region and subject may explain the behavioral RT effect.

## 3 RESULTS

### 3.1 Behavioral results

The group mean of the subject mean RTs for negative/neutral/positive distractors were 430/410/407 ms with standard errors of 14/12/12 ms, respectively. RTs were clearly dependent on distractor valence (F[2,86] = 12.764, *p* < .0005) (see Figure 1(b)). Subsequent one‐sided paired t‐tests revealed that mean(RT_neg_) > mean(RT_neu_) (t[43] = 3.997, *p* < .0005) with Cohen's d = 0.23 and d_z_ = 0.60. The mean difference was (19.4 ± 4.9 SEM [standard error of the mean]) ms. In average, valence category explained 2.6 (±0.7 SEM) % of the RT variance within a subject. It was also found that mean(RT_neg_) > mean(RT_pos_) (t[43] = 3.60, *p* < .0005). There was no significant effect mean(RT_pos_) > mean(RT_neu_) (t[43] = −1.28, *p* = .90).

Error rates were in average below 2 errors per category (see Figure S1). While the pattern of higher error rates for negative distractors is equivalent to the RT data, the effect of valence on error rate was not significant (F[2,86] = 1.266, *p* < .287).

### 3.2 fMRI results

**FIGURE 2:**
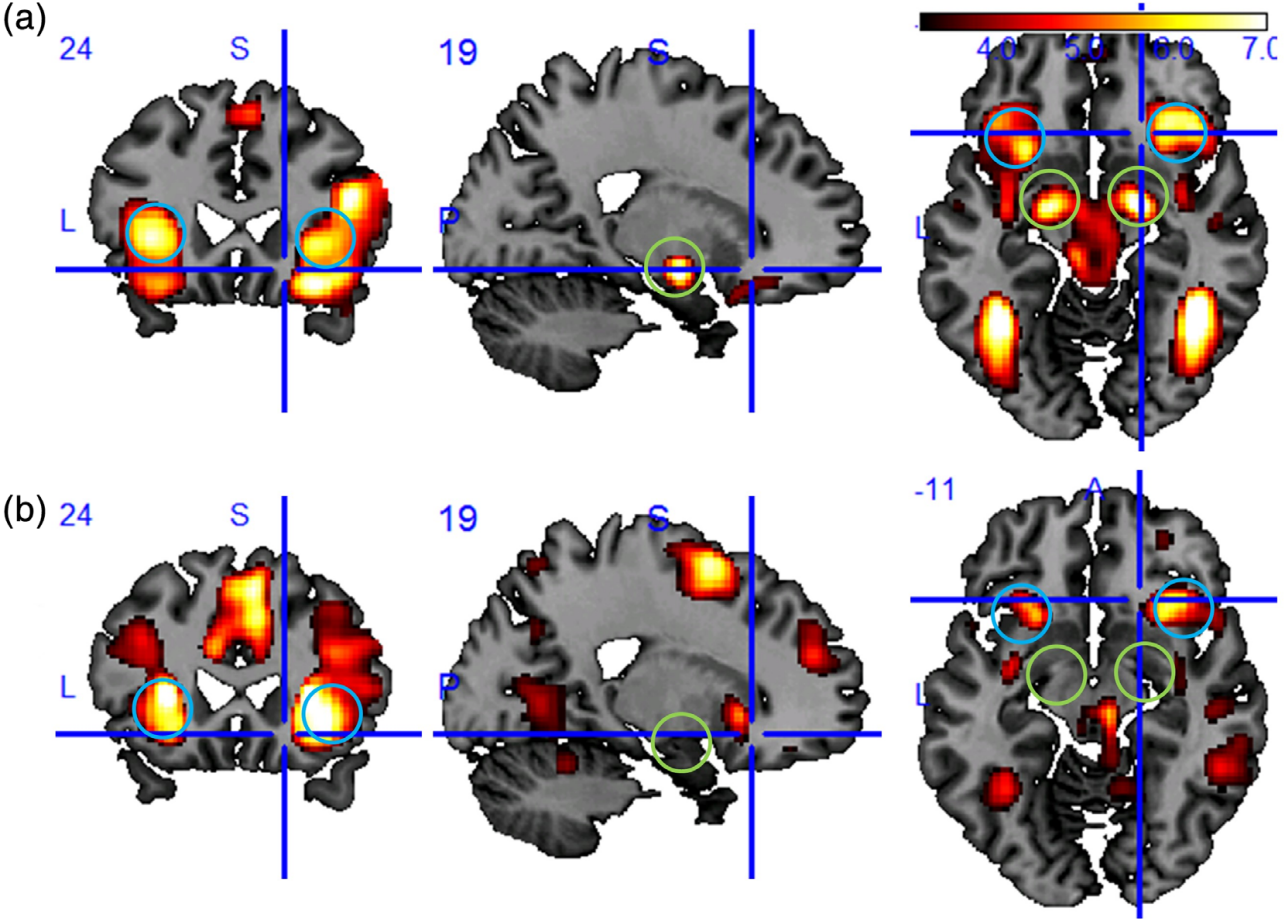
(a) Group activation maps at MNI = (19, 24, −11) (*N* = 40, *p* < .001 uncor.) for the contrast “negative > neutral distractor images” of the valence model (T[117] > 3.16) and (b) the parametric contrast “positive correlation with RT” for the RT model (T[39] > 3.31). Blue circles mark the anterior insula, green circles the amygdala

Based on our data, answers to the core questions of this paper are given in the right column of Table 1. The results of the Valence Model (#1) are shown in Figure 2(a) and Table 2(a) and address the question whether distractor valence has an effect on fMRI signals in amygdala and anterior insula. Both regions show bilaterally more signal for negative versus neutral distractors (“Negative > Neutral” contrast) for both the SVC (Table 2(a)) and the whole brain approach (Suppl. Figure S2 and Table S1), which demonstrates the robustness of the effect. Notably, there is also higher signal in the fusiform gyrus and the medial segment of the superior frontal gyrus (see discussion). The signal in the ROIs is not increased for positive stimuli when compared to neutral stimuli (“Positive > Neutral” contrast). Whole brain activations for all six valence contrasts are given in the Supplement at *p*(FWE) < .05 (minimum k = 50) (see Figures S2‐4 and Table S1‐2).

**TABLE 2:** Group activation results for a cluster threshold *p* > .001 and a small volume corrected *p*(FWE) < .05 using a bilateral amygdala and bilateral anterior insula mask for (a) the contrast “negative > neutral distractor images” of the valence model, (b) the parametric contrast “positive correlation with RT” for the RT model, and (c) a positive covariation of contrast “negative > neutral distractor images” with the equivalent RT distractor effect (ΔRT covariate model)

Cluster‐level			Peak			MNI coordinate			Anatomical region
*p* (FWE‐corr)	# voxels k	*p* (un‐corr.)	*p* (FWE‐corr.)	T	*p* (un‐corr.)	x	y	z {mm}	
(a) Group contrast: Negative > neutral									
.02	123	.103	0	7.51	0	20	‐6	−12	R Amy
.001	468	.004	0	7.25	0	−26	16	−16	L AIns
			0	6.63	0	−34	24	0	
			0	5.2	0	−36	6	−8	
.018	131	.094	0	7.09	0	−18	−6	−10	L Amy
.002	349	.011	0	6.74	0	34	26	0	R AIns
			0	6.18	0	28	20	−14	
			.001	4.88	0	40	20	−10	
			.007	4.25	0	38	8	−8	
(b) Group contrast: Positive correlation with RT									
.001	506	.008	0	8.02	0	36	16	2	R AIns
			0	7.42	0	30	26	6	
			0	7.38	0	34	26	−4	
.002	421	.014	0	7.93	0	−30	16	8	L AIns
			0	6.36	0	−28	24	−6	
			0	6.05	0	−26	20	−10	
(c) Group contrast: Positive covariate ΔRT									
.045	59	.224	.018	4.31	0	36	10	−8	R AIns
			.032	4.08	0	30	26	−6	

*Note: p*‐values smaller .0005 are quoted as 0.

The results of the RT Model (#2) are shown in Figure 2(b) and Table 2(b) and address the question whether voxel‐wise fMRI signal in amygdala and anterior insula are positively correlated with RT. While a positive correlation is found bilaterally in the anterior insula, no activity is seen in the amygdala even though a liberal SVC was used. A negative correlation with RT cannot be found in the ROIs. Whole brain results for both contrasts are given in the Supplement at *p*(FWE) < .05 (minimum k = 50) (see Figure S5 and Table S3).

The ΔRT Covariate Model (#3) addressed the question whether subjects with a higher RT distractor effect (ΔRT) also show a higher fMRI contrast in the ROIs and confirmed this hypothesis in the right anterior insula (Table 2(c) and Suppl. Figure S6) but not in the other regions.

**FIGURE 3:**
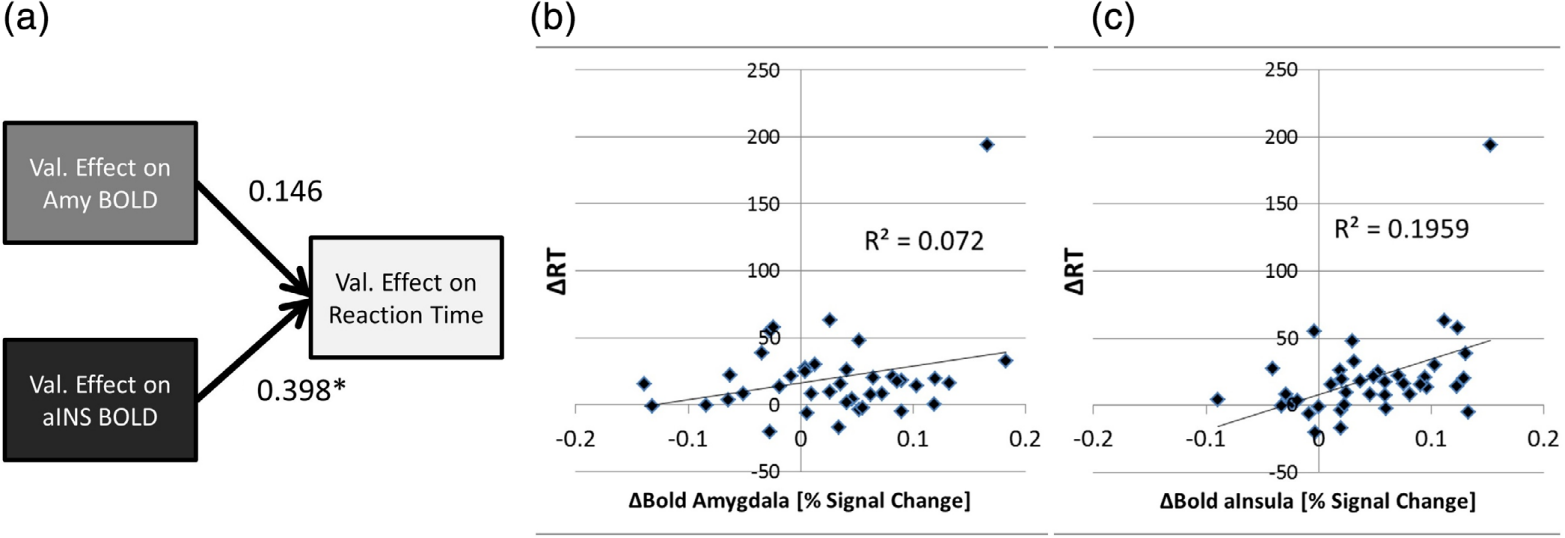
Linear regression of the RT distractor effect (ΔRT = mean(RTneg) – mean(RTneu)) as a function of the fMRI valence effects in amygdala and anterior insula shows a significant (* *p* < .05) correlation of the anterior insula activity with ΔRT, while the amygdala activity is not significantly related to ΔRT (standardized fitting coefficients are given in (a), *N* = 40). (B) and (C) show ΔRT as a function (bi‐variate) of the amygdala BOLD effect and the anterior insula BOLD effect, respectively. Linear trend lines with the corresponding R2‐values are also shown

Similarly, considering ΔRT as a dependent variable of the BOLD “Negative‐Neutral” contrasts in the amygdala and insula in the ΔRT versus BOLD Model (#4) reveals a significant effect of the anterior insula (t[37] = 2.598, *p* = .013) but not of the amygdala (t[37] = 0.952, *p* = .347). The results of this analysis are shown in Figure 3(a). An analysis without the outlier in ΔRT in Figure 3(a), does not change this result in a major way (effect of the insula: t(36) = 2.316, *p* = .026; amygdala: t(36) = 0.405, *p* = .688). Conventional, bi‐variate correlation plots of ΔRT versus BOLD contrast are also shown for both the amygdala (Figure 3(b)) and the insula (Figure 3(C)). Only the correlation with the anterior insula is significant. Anterior insula activity explains 20% of the variance in ΔRT (*R* ^2^ = .196, *p* = .004). There is a positive correlation (bivariate) between the fMRI valence effect in the two ROIs (*R* ^2^ = .095, *p* = .027, 1‐sided), which is expected if valence is a common source of the variance in both regions.

## 4 DISCUSSION

Monitoring and evaluating background or task‐irrelevant stimuli is a pivotal aspect of cognitive control, and, at times, our life may depend on it. Thus, it is important to understand how the brain accomplishes this task. In this study, we examined models of attentional capture by emotional stimuli that regard the insula or the amygdala or both as causally responsible for the capture of cognitive resources as measured by RT slowing. Using fMRI, we investigated trial‐by‐trial correlations of BOLD signal from these regions with RTs. While we found evidence supporting the involvement of the insula, we did not find a significant correlation with RTs for the amygdala, despite a liberal SVC approach to minimize false negative errors, and a large amount of literature implicating this region in the capture process. In addition, we found that subjects that exhibit a stronger emotional capture effect also show a stronger BOLD signal for negative distractors as compared to neutral distractors in the right anterior insula but not in the amygdala. These results suggest that the insula may be an important mediator of RT slowing while no evidence was found that the amygdala is involved.

Our work emphasizes the difference between plausibility and evidence. While it is plausible that the amygdala is involved in RT slowing through emotional distraction, there is currently no convincing neuroimaging evidence in humans to support this conclusion. For the insula, on the other hand, we could demonstrate both valence category‐related activity as well as RT‐related activity. Our conclusions from these findings are two‐fold: First, we advocate that models of emotional capture should take the anterior insula into account as a potentially important player in addition to the much more frequently discussed amygdala. We advocate, that the anterior insula needs to be considered as an integral part of the “Hot Emotional System” as described by Dolcos et al. (Dolcos et al., 2011). The literature relating the insula to both emotions and motor responses is vast (Gogolla, 2017; Uddin, Nomi, Hebert‐Seropian, Ghaziri, & Boucher, 2017). However in the context of emotional capture, we share the sentiment expressed by Namkung, Kim and Sawa in their opinion paper “The Insula: An Underestimated Brain Area in Clinical Neuroscience, Psychiatry, and Neurology” (Namkung, Kim, & Sawa, 2018). An interesting model of anterior insula function in this context could be, for example, that the insula, which has been described as a network hub (Uddin et al., 2017), serves as a distributor and potential amplifier of emotional signals, potentially originating in the amygdala and possibly biased by associations with visceral, emotional perceptions (Chang et al., 2013).

Second, while we do not intend to question the importance of the amygdala for emotional responses, we emphasize that more sensitive experiments are needed to firmly establish its involvement in RT slowing or other behavioral effects of emotional capture. Ways to accomplish this include increasing the RT distractor effect by individualized stimulus selection and using clinical populations, reducing RT variability by EEG‐based, real‐time triggering of stimulus presentation, improved fMRI acquisitions through multi‐band EPI, and optimized data modeling of trial‐by‐trial responses. A better understanding of both insula and amygdala function in the context of normal and pathological emotional processing may even aid the development of targeted therapies such as fMRI neurofeedback (Marxen et al., 2016).

The ACES task employed in this study was optimized based on typical emotional capture tasks in the field and produces an emotional capture effect as measured by a slowing of RTs. We also demonstrated that this task elicits a higher BOLD signal for negative distractors than for neutral distractors in the amygdala and the anterior insula. The results of the whole‐brain analysis of the “Negative > Neutral” contrast are similar to Mitchell et al. (Mitchell et al., 2008) and consistent with the hot emotional system (HotEmo) as described by Dolcos et al. and Iordan et al. (Dolcos et al., 2011; Iordan et al., 2013) showing the bilateral fusiform gyrus (or occipitotemporal cortex), the bilateral amygdala, and two (bilateral) large clusters covering parts of the orbital frontal gyrus (OFC), the insula, and the ventrolateral prefrontal cortex (vlPFC). Activation in the OFC and the vlPFC was stronger on the right hemisphere (see Figure 2(a) and Suppl. Figure S2, Table S1). The cold executive system (ColdEx) can also be identified in the “Negative < Neutral” contrast (see Suppl. Figure S2, Table S1) in the right angular gyrus (AnG), also referred to as lateral parietal cortex (LPC) and the right Frontal Pole (FP), also referred to as dorsolateral PFC (dlPFC). Activation in the contralateral “cold” regions are apparent at lower significance thresholds. Dolcos et al. (Dolcos et al., 2011), focusing on working memory tasks, also consider the middle temporal lobe a part of the Cold Ex system, which we do not see for our ACES task.

Beyond the two ROIs relevant for our research question, the parametric RT contrast shows a number of regions that are not part of the “Negative > Neutral” contrast and have consequently not been considered as regions‐of‐interest for our hypothesis‐driven approach. These regions include in particular regions related to sensori‐motor activity, that is, the primary sensori‐motor cortex, the supplementary motor area and parietal regions (see Table S3). It is, however, not obvious why sensori‐motor regions are more active for longer reaction times and, also, why there is no activation for shorter reaction times. In most motor experiments, block designs of motor activity (e.g., finger tapping) are used to induce brain activity; reaction times are not considered. We are using an event‐related design and RTs are crucial for us as a behavioral read‐out. Though only of peripheral relevance to our study, some interesting observations can be made with respect to the brain activity correlated with RT (RT contrast) when comparing it to the group contrast “All Onsets > 0 (Baseline)” from Model 1 (onset contrast), which shows strong activation of the motor system (among others) due to the button presses (right hand) following each onset. The RT contrast cluster around the left central sulcus (primary sensori‐motor cortex, contra‐lateral to the active hand) is centered on the post‐central, somatosensory region and more posterior than the onset contrast, which clearly includes the pre‐central motor cortex (see Figure S7). Thus, slower reactions are associated with stronger sensory activation of the finger/hand‐region but not with stronger (primary) motor activity. Looking closer at the supplementary motor area (SMA), the activation of the RT contrast is focussed more anteriorly compared to the onset contrast and spreading laterally (Figure S8). This may be an indication that higher BOLD signals for longer reaction times in the motor system are the result of a longer phase of motor planning in the pre‐motor cortex with a different signal distribution as compared to the overall motor control (onset contrast). Consequently, no activation is observed that is larger for faster reactions. This explanation may also apply to the parietal cortex, which is active in the RT contrast partially overlapping the onset contrast (Figure S9). Furthermore, the dorsal anterior cingulate cortex (dACC) is involved in motor tasks (Amiez & Petrides, 2014) but also considered an important part of the salience network (Seeley et al., 2007). In our data, the dACC is active for the RT contrast and shows activation connected to the supplementary motor area and a pronounced cluster in the more anterior section of the dACC while the onset contrast is much weaker in dACC as compared to the supplementary motor area (see Figure S10). This may indicate that dACC is also involved in motor planning. Importantly, the dACC shows no activation in the valence contrast. The valence contrast activates actually more superficial regions of the medial superior frontal gyrus (see Figure S11). Lastly, the insula cortex activation in the RT contrast is regionally consistent with the onset contrast (see Figure S12). Thus, the three major regions of the salience network anterior insula, amygdala and dACC are clearly playing different roles in our task. While insula and amygdala are sensitive to valence, the dACC is not. Instead, the dACC is more active for reactions that take more time while the amygdala is not. Only the anterior insula is active in both contrasts. Therefore, it is a prime candidate for mediating the distractor effect.

In our results, we did not see a significant effect of valence on error rates. This may be a consequence of utilizing a very simple RT task with very few errors or the exclusion of subjects with a high error rate. Limited pilot studies indicated a larger valence effect on RT, which was the focus of our study, for simpler tasks, possibly due to interference at an automatic level of processing and less variance in the RT data compared to cognitively more demanding tasks. We also saw no contrast‐of‐interest in fMRI signal and no RT difference between positive and neutral images. This is not very surprising because effects for this contrast are known to be smaller than for the negative versus neutral contrast (see for example, (Mitchell et al., 2008)) especially when the positive images are not predominantly of sexual content as in our study. In line with this, positive images also had a lower mean arousal rating than negative images (see Table S4). The positive contrast was not of primary interest for this analysis but included in the paradigm because a potential effect could have been informative for future studies in clinical populations.

Some limitations of our study typical for fMRI need to be noted. Our findings from the RT model cannot be regarded as sufficient to conclude that valence related RT changes are mediated by the insula within subjects. In fact, valence category explains in average only about 3% of the within‐subject RT variance. Consequently, removing arousal and valence effects from the parametric RT contrast has almost no impact on the activation pattern (data not shown) indicating that the insula parametric RT contrast is driven by general RT variance and may be independent of the emotional distractors. This could also be the reason why no correlation with RT can be identified within the amygdala, that is, there is a sensitivity issue resulting in a type 2 error (false negative) if amygdala activity would only be related to such a small part of RT variance. Another option is that the contrast “Negative > Neutral” is driven by other image features than those that drive the RT effect. Horstmann et al. have reported a similar effect that the flanker‐effect asymmetry with affective faces cannot be unambiguously attributed to emotional differences of the stimuli but may be due to purely perceptual differences (Horstmann, Borgstedt, & Heumann, 2006).

We should also consider the possibility that blood flow / oxygenation based imaging methods such as fMRI are not effective to show the causal role of the amygdala for RT slowing because the amygdala may not influence RTs through mechanisms resulting in altered “BOLD activity” but through otherwise different “activity”. An interesting experimental approach to study this are intracranial electrodes. Sonkusare et al. reported, for example, that emotional valence modulated connectivity between the amygdala and the temporal pole in six epilepsy patients using such electrodes (Sonkusare et al., 2018). Unfortunately, noninvasive neurostimulation techniques, which are suitable for testing causal relationships, are not yet sufficiently mature to modulate subcortical structures such as the amygdala.

Furthermore, it should be noted that our paradigm does not show the emotional distractor and the task cue concurrently in the same frame like many other attentional capture paradigms (Carretie, 2014). Consequently, we are not studying the direct competition of two visual stimuli for attention but rather the temporary capture of attention or cognitive resources by a salient stimulus, a phenomenon that is similar to the well‐known attentional blink effect (Shapiro, Raymond, & Arnell, 1997). The reason for this choice of paradigm was that we were not able to produce a similar valence effect on RT (or measure of emotional reactivity) in healthy participants using concurrent stimuli. In fact, in a study with concurrent stimuli, we found no significant RT difference between negative versus neutral distractors in *N* = 136 subjects (Pilhatsch et al., 2014b). Additionally, our distractors are shown before and after the task cue. Thus, we cannot be sure at which level of executing the task the distraction effect occurs. For example, the preattention/evaluation phase may not be responsible for the RT slowing. Instead, displaying the distractor images after the task cue may effect motor preparation and execution. More clarity on this issue requires further experimental studies, such as a behavioral experiment without displaying the post‐cue distractors. For this study, we simply adopted the timing from Mitchell et al. (Mitchell et al., 2008) and believe that showing the distractor image for 400 ms after the task cue is necessary to produce an fMRI valence contrast because images that are shown for 200 ms only would not produce a large enough BOLD response.

Lastly, the “Negative > Neutral” contrast strongly activates not only amygdala and anterior insula but also the fusiform gyrus. Boubela et al. (Boubela et al., 2015) have pointed out that the vein that likely drains the fusiform region, the basal vein of Rosenthal, passes closely by the amygdala and that this may cause a BOLD signal that would not reflect neural activity of the amygdala. We cannot exclude this effect in our data, however, our conclusions would largely be unchanged given that this effect would further question the amygdala activation with the observed insula effects remaining.

## CONFLICT OF INTEREST

All authors declare no competing interests.

## AUTHOR CONTRIBUTIONS

All authors have contributed to the drafting of the manuscript, have agreed to its content, and have agreed to be accountable for all aspects of this work. **All authors** were involved in: Investigation, Writing –Review & Editing. Additionally, **Michael Marxen:** Conceptualization, Methodology, Software, Validation, Formal analysis, Data Curation, Writing –Original Draft, Visualization, Supervision, Project Administration, Funding acquisition; **Mark J. Jacob:** Conceptualization, Methodology, Software, Formal analysis, Data Curation, Project Administration; **Lydia Hellrung:** Methodology, Software, Validation, Formal analysis; **Philipp Riedel:** Methodology, Formal analysis; **Michael N. Smolka:** Conceptualization, Methodology, Supervision, Project Administration, Funding acquisition.

## Supporting information


**Appendix** **S1**: Supporting Information

## Data Availability

Statistical maps, SPSS and Matlab code are available within the OSF project “SFB940/1‐A7: Volitional Control of Brain Activity: Effects of Neurofeedback on Emotional Reactivity” (Marxen, 2020b) from the authors upon request in the component “Manuscript: Amygdala and Insula in Attentional Capture by Emotional Stimuli” (Marxen, 2020a). Original DICOM data and EPI images are currently NOT open access because the issue of “fingerprint information” within MRI‐data is a topic of ongoing, ethical debate in Germany. It is unclear whether this data can legally be considered “anonymized”. Discussions to establish institutional guidelines are ongoing.

## Endnote

^1^
Atlas is based on labels provided by http://Neuromorphometrics.com/ and MRI scans by http://www.oasis-braIns.org .
