# Supplementary material for: Questioning the role of amygdala and insula in an attentional capture by emotional stimuli task
Source: Hum Brain Mapp. 2020 Nov 20;42(5):1257–67. doi: 10.1002/hbm.25290 (PMC7927307; doi:10.1002/hbm.25290)
Supplement: Supplementary file 1 — Appendix S1: Supporting Information [file HBM-42-1257-s001.docx]

**Supplemental Information**

for

**Questioning the Role of Amygdala and Insula in a Choice Reaction Time Task with Emotional Distractors**

Michael Marxen^1^, Mark J. Jacob^1^, Dirk K. Müller^1^, Lydia Hellrung^1^, Philipp Riedel^1^, and Michael N. Smolka^1^

^1^ Department of Psychiatry and Neuroimaging Center, Technische Universität Dresden, 01197 Dresden, Germany

Abbreviations:

R - Right, L - Left

AIns - Anterior Insula

Amy - Amygdala

AnG - Angular Gyrus

Cerebral WM - Cerebral White Matter

FO - Frontal Operculum

FP - Frontal Pole

FuG - Fusiform Gyrus

IOG - Inferior Occipital Gyrus

ITG - Inferior Temporal Gyrus

MFG - Middle Frontal Gyrus

MSFG - Superior Frontal Gyrus Medial Segment

MTG - Middle Temporal Gyrus

OpIFG - Opercular part of the Inferior Frontal Gyrus

OrIFG - Orbital part of the Inferior Frontal Gyrus

PCu - Precuneus

PoG - Postcentral Gyrus

POrG - Posterior Orbital Gyrus

PT - Planum Temporale

SMG - Supramarginal Gyrus

SMC – Supplementary Motor Cortex

SPL - Superior Parietal Lobule

STG - Superior Temporal Gyrus

TMP - Temporal Pole


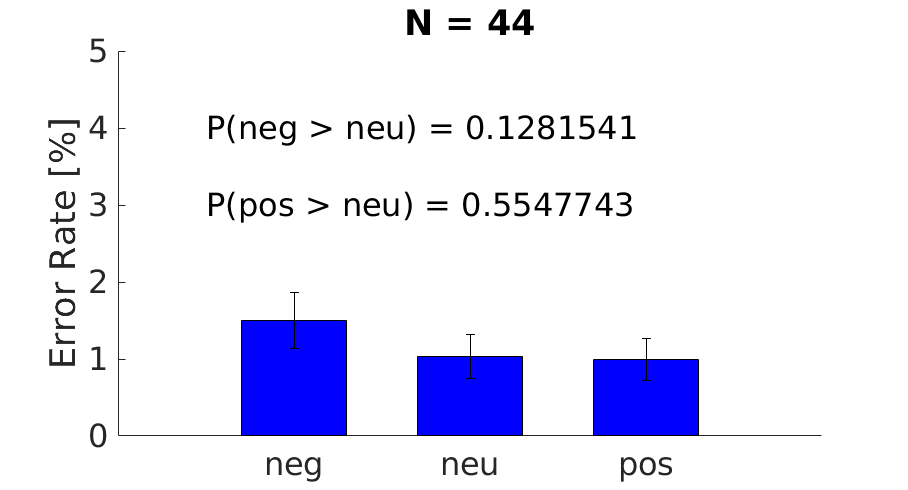


Figure S1: Effect of valence category on mean error rates with standard error (N=44). The effect of valence was not significant (F(2,86) = 1.266, P = 0.29). P-values for one-sided t-tests are given in the figure.


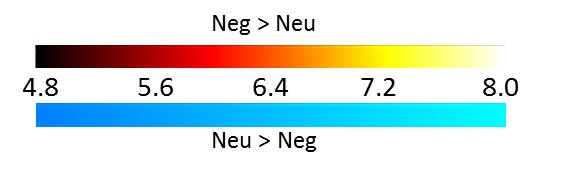


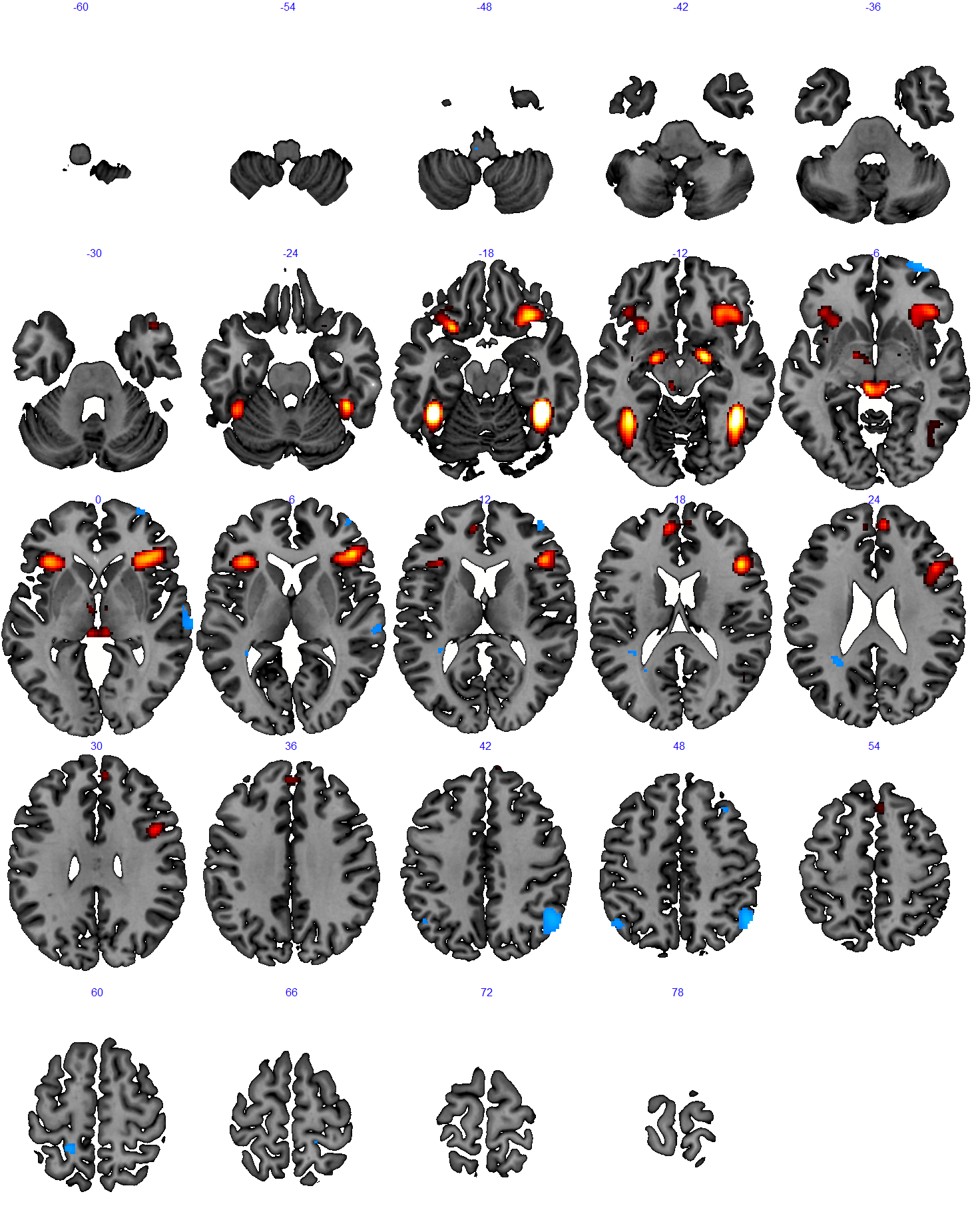


Figure S2: Group activation maps (N=40, P(FWE) < 0.05) for the contrast „Negative > Neutral” and “Neutral > Negative” distractor images of the Valence Model (T(117) > 4.74).

| Cluster-Level | | | Peak | | | MNI coordinate | | | Anatomical Region |
| --- | --- | --- | --- | --- | --- | --- | --- | --- | --- |
| P(FWE-corr) | # voxels k | P(un-corr.) | P(FWE-corr.) | T | P(un-corr.) | x | y | z {mm} |  |
| Group Contrast: Negative > Neutral | | | | | | | | | |
| 0 | 798 | 0 | 0 | 11 | 0 | 42 | -48 | -16 | R FuG |
| 0 | 636 | 0 | 0 | 9.75 | 0 | -40 | -48 | -18 | L FuG |
| 0 | 143 | 0.003 | 0 | 7.71 | 0 | 18 | -6 | -10 | R Cerebral WM* |
| 0 | 1819 | 0 | 0 | 7.37 | 0 | 32 | 26 | -16 | R Cerebral WM** |
|  |  |  | 0 | 7.18 | 0 | 46 | 24 | 20 | R OpIFG |
|  |  |  | 0 | 7.12 | 0 | 44 | 32 | 4 | R Cerebral WM |
| 0 | 863 | 0 | 0 | 7.25 | 0 | -26 | 16 | -16 | L POrG** |
|  |  |  | 0 | 7.07 | 0 | -36 | 26 | 2 | L FO |
|  |  |  | 0.002 | 5.64 | 0 | -38 | 30 | -10 | L Cerebral WM** |
| 0 | 183 | 0.001 | 0 | 7.2 | 0 | -16 | -6 | -10 | L Cerebral WM* |
|  |  |  | 0.006 | 5.3 | 0 | -6 | -10 | -2 | L Thalamus |
| 0 | 310 | 0 | 0 | 7.02 | 0 | -4 | -30 | -6 | Brain Stem |
|  |  |  | 0 | 6.59 | 0 | 4 | -30 | -4 | Brain Stem |
| 0 | 317 | 0 | 0 | 6.23 | 0 | 8 | 54 | 24 | R MSFG |
|  |  |  | 0 | 6.22 | 0 | -8 | 52 | 18 | L MSFG |
|  |  |  | 0.007 | 5.29 | 0 | 0 | 46 | 36 | No Label |
| Group Contrast: Negative < Neutral | | | | | | | | | |
| 0 | 274 | 0 | 0 | 6.42 | 0 | 50 | -58 | 46 | R AnG |
| 0.003 | 50 | 0.058 | 0.001 | 5.72 | 0 | -20 | -46 | 62 | L SPL |
| 0.001 | 73 | 0.026 | 0.002 | 5.61 | 0 | 32 | 64 | -4 | R FP |
| 0 | 104 | 0.01 | 0.003 | 5.53 | 0 | 70 | -22 | 2 | R STG |
|  |  |  | 0.017 | 5.05 | 0 | 64 | -8 | -2 | R STG |
|  |  |  | 0.018 | 5.03 | 0 | 60 | -28 | 8 | R Cerebral WM |
| 0.001 | 78 | 0.022 | 0.004 | 5.45 | 0 | -28 | -50 | 22 | L Cerebral WM |
|  |  |  | 0.013 | 5.11 | 0 | -36 | -42 | 18 | L Cerebral WM |
|  |  |  | 0.018 | 5.03 | 0 | -34 | -40 | 10 | L Cerebral WM |

Table S1: Whole-brain group activation results for P(FWE) > 0.05 (minimum cluster extent threshold 50 voxel) for the contrasts „Negative > Neutral“ and “Negative < Neutral” of the Valence Model as visualized in Fig. S2. Clusters covering part of the amygdala are marked with “*” and clusters covering part of the anterior insula are marked with “**”.


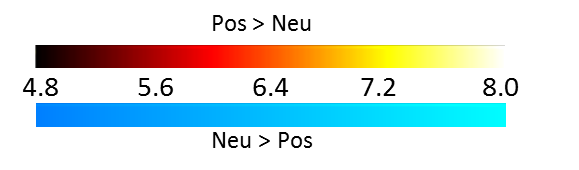


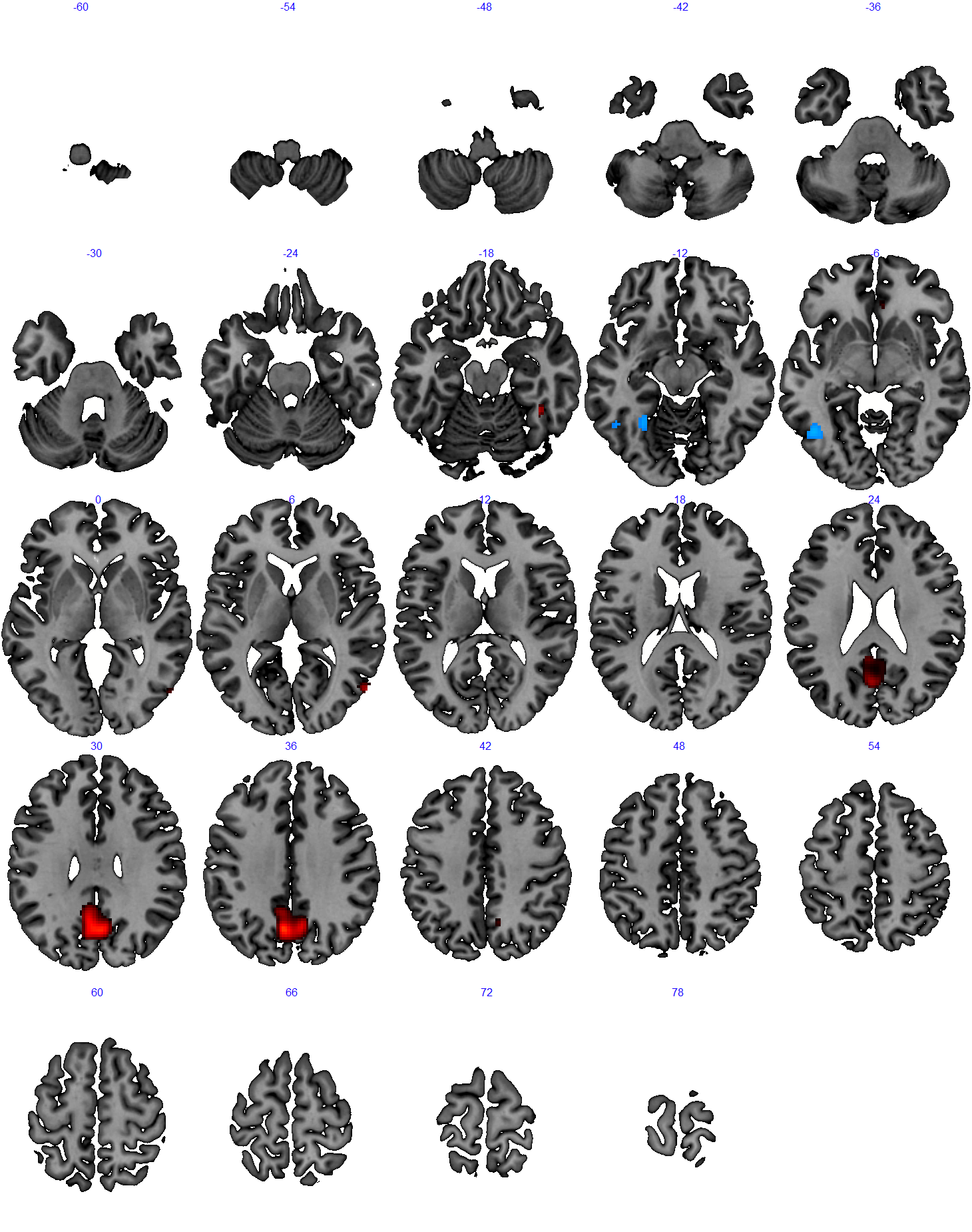


Figure S3: Group activation maps (N=40, P(FWE) < 0.05) for the contrast „Positive > Neutral” and “Neutral > Positive” distractor images of the Valence Model (T(117) > 4.74).


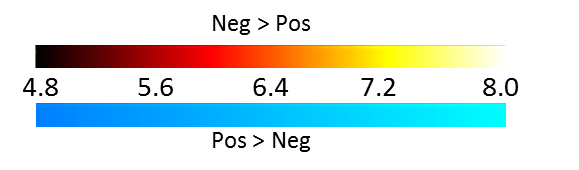


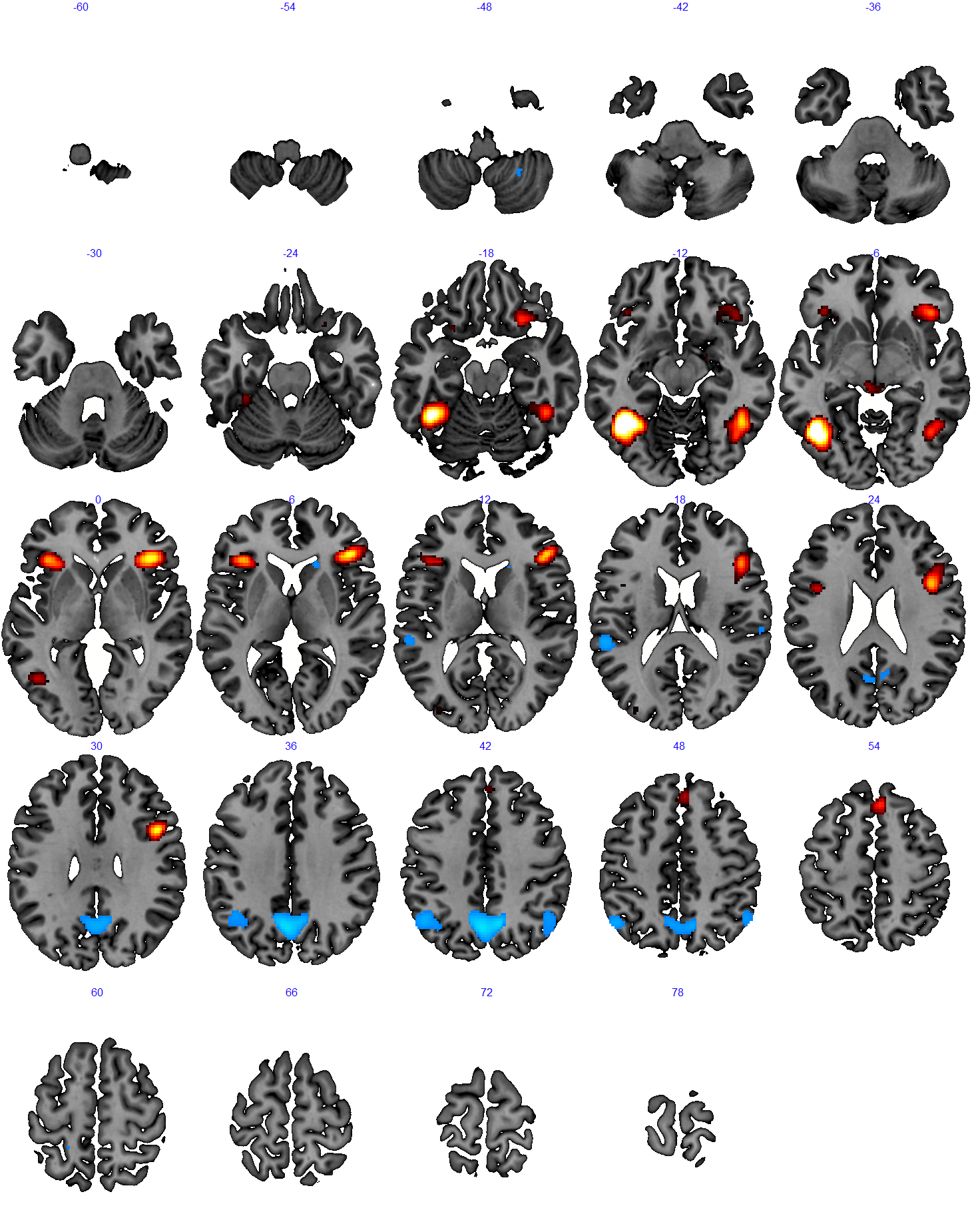


Figure S4: Group activation maps (N=40, P(FWE) < 0.05) for the contrast „Negative > Positive” and “Positive > Negative” distractor images of the Valence Model (T(117) > 4.74).

| Cluster-Level | | | Peak | | | MNI coordinate | | | Anatomical Region |
| --- | --- | --- | --- | --- | --- | --- | --- | --- | --- |
| P(FWE-corr) | # voxels k | P(un-corr.) | P(FWE-corr.) | T | P(un-corr.) | x | y | z {mm} |  |
| Group Contrast: Positive > Neutral | | | | | | | | | |
| 0 | 1038 | 0 | 0 | 6.31 | 0 | -6 | -64 | 34 | L PCu |
|  |  |  | 0 | 6.11 | 0 | 4 | -66 | 30 | R PCu |
| 0.003 | 50 | 0.058 | 0.001 | 5.72 | 0 | 56 | -70 | 4 | R IOG |
| Group Contrast: Positive < Neutral | | | | | | | | | |
| 0 | 111 | 0.008 | 0 | 5.96 | 0 | -46 | -62 | -6 | L Cerebral WM |
| 0.002 | 58 | 0.043 | 0.001 | 5.71 | 0 | -28 | -56 | -10 | L FuG |
| Group Contrast: Negative > Positive | | | | | | | | | |
| 0 | 1278 | 0 | 0 | 9.51 | 0 | -44 | -58 | -10 | L ITG |
| 0 | 1627 | 0 | 0 | 7.49 | 0 | 44 | 32 | 8 | R Cerebral WM |
|  |  |  | 0 | 7.42 | 0 | 44 | 10 | 28 | R OpIFG |
|  |  |  | 0 | 7.37 | 0 | 40 | 28 | 0 | R FO |
| 0 | 568 | 0 | 0 | 7.19 | 0 | 48 | -56 | -10 | R ITG |
|  |  |  | 0 | 6.96 | 0 | 44 | -48 | -16 | R FuG |
|  |  |  | 0 | 6.79 | 0 | 42 | -64 | -10 | R Cerebral WM |
| 0 | 498 | 0 | 0 | 6.98 | 0 | -36 | 26 | 2 | L FO |
|  |  |  | 0.006 | 5.34 | 0 | -40 | 28 | -10 | L Cerebral WM |
| 0 | 203 | 0.001 | 0 | 6.39 | 0 | 4 | 30 | 52 | R MSFG |
| 0.002 | 68 | 0.03 | 0.001 | 5.68 | 0 | -44 | 6 | 24 | L OpIFG |
| 0.002 | 67 | 0.032 | 0.003 | 5.49 | 0 | -4 | -28 | -6 | Brain Stem |
| Group Contrast: Negative < Positive | | | | | | | | | |
| 0 | 993 | 0 | 0 | 7.52 | 0 | -4 | -64 | 40 | L PCu |
|  |  |  | 0 | 7.08 | 0 | 10 | -60 | 42 | R Cerebral WM |
| 0 | 309 | 0 | 0 | 6.05 | 0 | -46 | -60 | 44 | L AnG |
| 0 | 112 | 0.008 | 0 | 5.96 | 0 | -56 | -34 | 16 | L PT |
| 0 | 168 | 0.002 | 0.002 | 5.59 | 0 | 50 | -56 | 46 | R AnG |
|  |  |  | 0.014 | 5.09 | 0 | 46 | -66 | 42 | R AnG |

Table S2: Whole-brain group activation results for P(FWE) > 0.05 (minimum cluster extent threshold 50 voxel) for the contrasts „Positive > Neutral“, “Postive < Neutral”, “Negative > Positive”, and “Negative < Positive” of the Valence Model as visualized in Figs. S3-4.


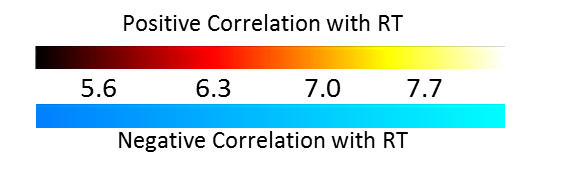


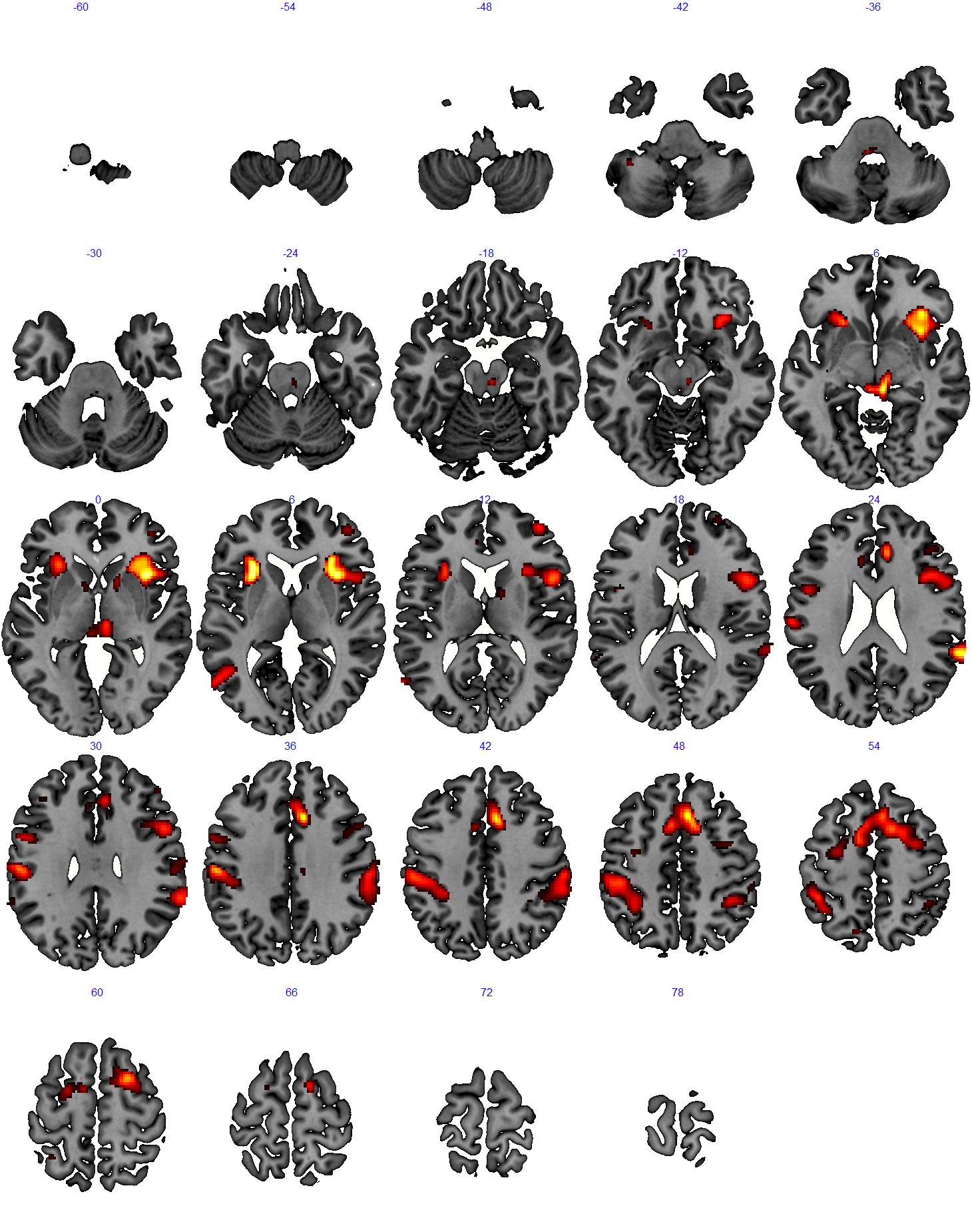


Figure S5: Group activation maps (N=40, P(FWE) < 0.05, minimum cluster extent threshold 50 voxel) for the contrast „Positive Correlation with RT“ of the RT Model (T(39) > 5.20).
There were no significant activations for “Negative Correlation with RT”.

| Cluster-Level | | | Peak | | | MNI coordinate | | | Anatomical Region |
| --- | --- | --- | --- | --- | --- | --- | --- | --- | --- |
| P(FWE-corr) | # voxels k | P(un-corr.) | P(FWE-corr.) | T | P(un-corr.) | x | y | z {mm} |  |
| Group Contrast: Positive Correlation with RT | | | | | | | | | |
| 0 | 2150 | 0 | 0 | 8.02 | 0 | 36 | 16 | 2 | R AIns |
|  |  |  | 0 | 7.48 | 0 | 28 | 22 | 6 | R Cerebral WM |
|  |  |  | 0 | 7.47 | 0 | 36 | 26 | -6 | R OrIFG |
| 0 | 577 | 0 | 0 | 7.93 | 0 | -30 | 16 | 8 | L AIns |
|  |  |  | 0.001 | 6.75 | 0 | -24 | 20 | -6 | L Cerebral WM |
| 0 | 2291 | 0 | 0 | 7.61 | 0 | 8 | 18 | 38 | R SMC |
|  |  |  | 0 | 7.51 | 0 | 6 | 20 | 50 | R SMC |
|  |  |  | 0 | 6.96 | 0 | 22 | 8 | 60 | R SFG |
| 0 | 382 | 0 | 0 | 7.55 | 0 | 6 | -30 | -6 | Brain Stem |
|  |  |  | 0.001 | 6.67 | 0 | -4 | -30 | -4 | Brain Stem |
|  |  |  | 0.005 | 6.09 | 0 | 4 | -24 | -18 | Brain Stem |
| 0 | 1214 | 0 | 0 | 7.42 | 0 | 64 | -42 | 26 | R SMG |
|  |  |  | 0.001 | 6.67 | 0 | 60 | -24 | 40 | R SMG |
|  |  |  | 0.001 | 6.53 | 0 | 58 | -34 | 40 | R SMG |
| 0 | 1391 | 0 | 0 | 7.09 | 0 | -56 | -20 | 36 | L PoG |
|  |  |  | 0.001 | 6.62 | 0 | -52 | -28 | 44 | L SMG |
|  |  |  | 0.001 | 6.54 | 0 | -44 | -30 | 46 | L PoG |
| 0 | 225 | 0.001 | 0.002 | 6.45 | 0 | -56 | -64 | 8 | L MTG |
| 0 | 215 | 0.001 | 0.002 | 6.32 | 0 | 40 | 52 | 12 | R MFG |
|  |  |  | 0.009 | 5.84 | 0 | 40 | 48 | 2 | R MFG |
|  |  |  | 0.015 | 5.67 | 0 | 30 | 58 | 16 | R SFG |
| 0 | 253 | 0 | 0.003 | 6.25 | 0 | -50 | 4 | 26 | L PrG |
| 0.002 | 67 | 0.043 | 0.02 | 5.55 | 0 | 46 | 38 | 28 | R MFG |
|  |  |  | 0.02 | 5.55 | 0 | 42 | 34 | 22 | R Cerebral WM |

Table S3: Whole-brain group activation results for P(FWE) > 0.05 (minimum cluster extent threshold 50 voxel) for the contrasts „Positive Correlation with RT“ of the RT Model as shown in Fig. S5. There were no significant activations for “Negative Correlation with RT”.


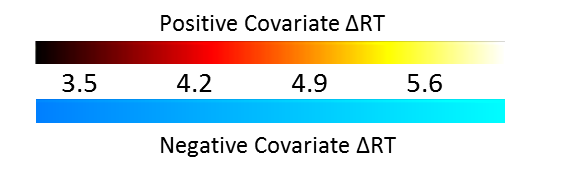


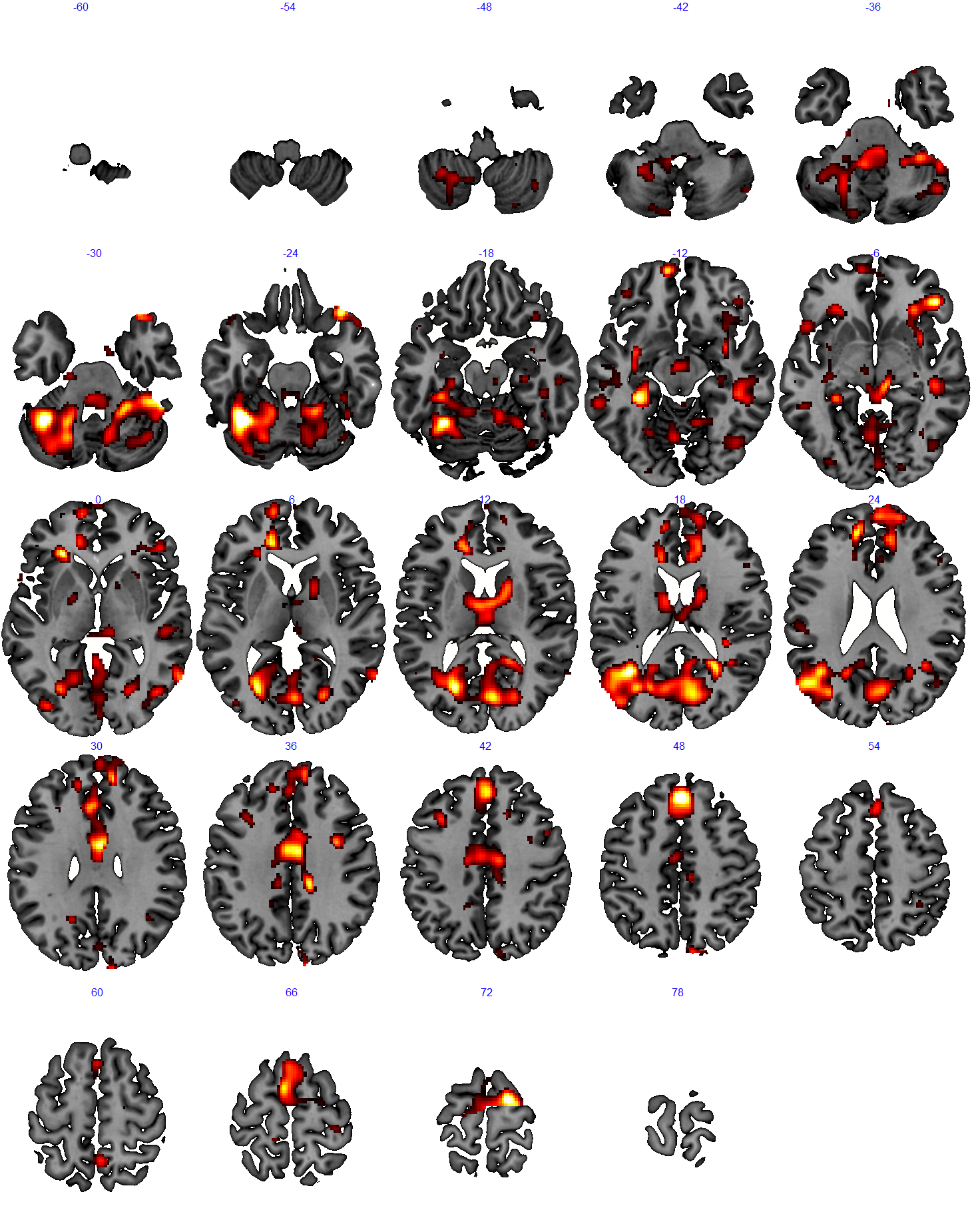


Figure S6: Group activation maps (N=40, P(uncor.) < 0.001) for a „Positive Covariate ∆RT“ of the ∆RT Covariate Model (T(38) > 3.32). There were no significant activations for a “Negative Covariate ∆RT”. Only the cluster in the right anterior insula is significant when using SVC with the amygdala-insula mask and a peak P(FWE) < 0.05 (see Table 1 within the main manuscript).


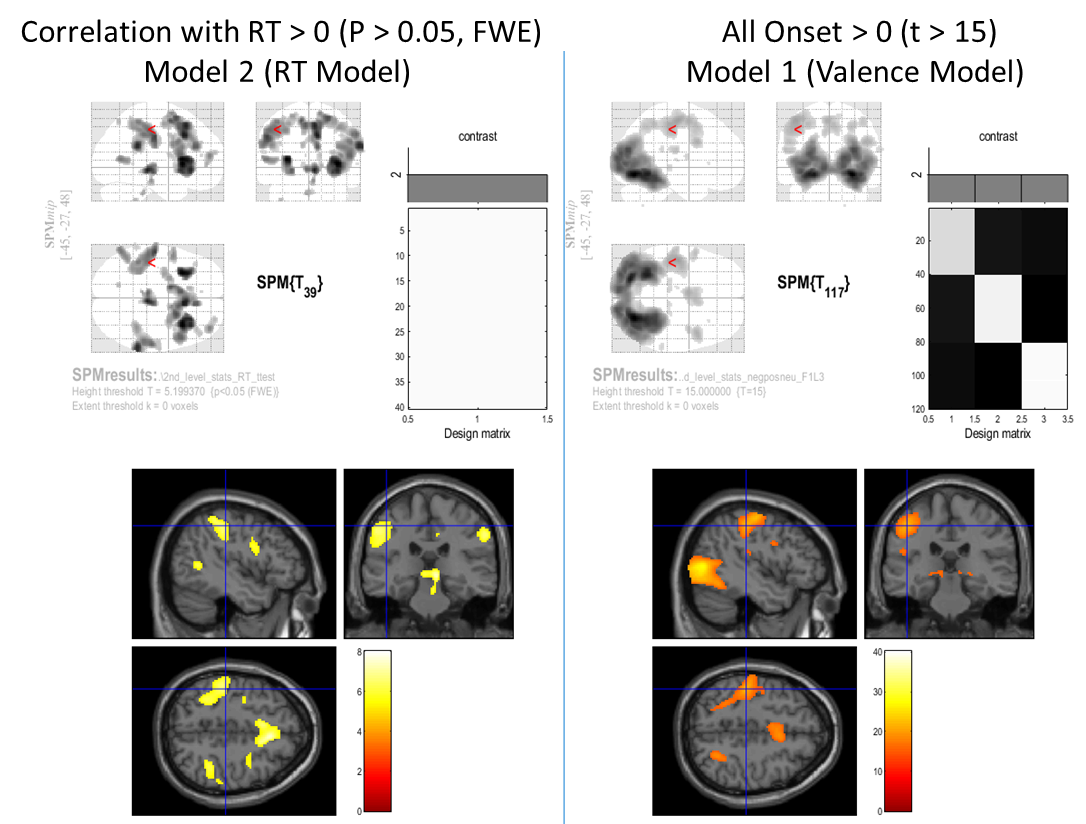


Figure S7: Comparing the parametric RT contrast from Fig. S5 (P < 0.05 FWE) with the contrast “All Onsets > Baseline” from Model 1 (Valence Model) (T-value > 15 for illustrative purposes) centering on the central sulcus, i.e. the primary sensori-motor cortex.


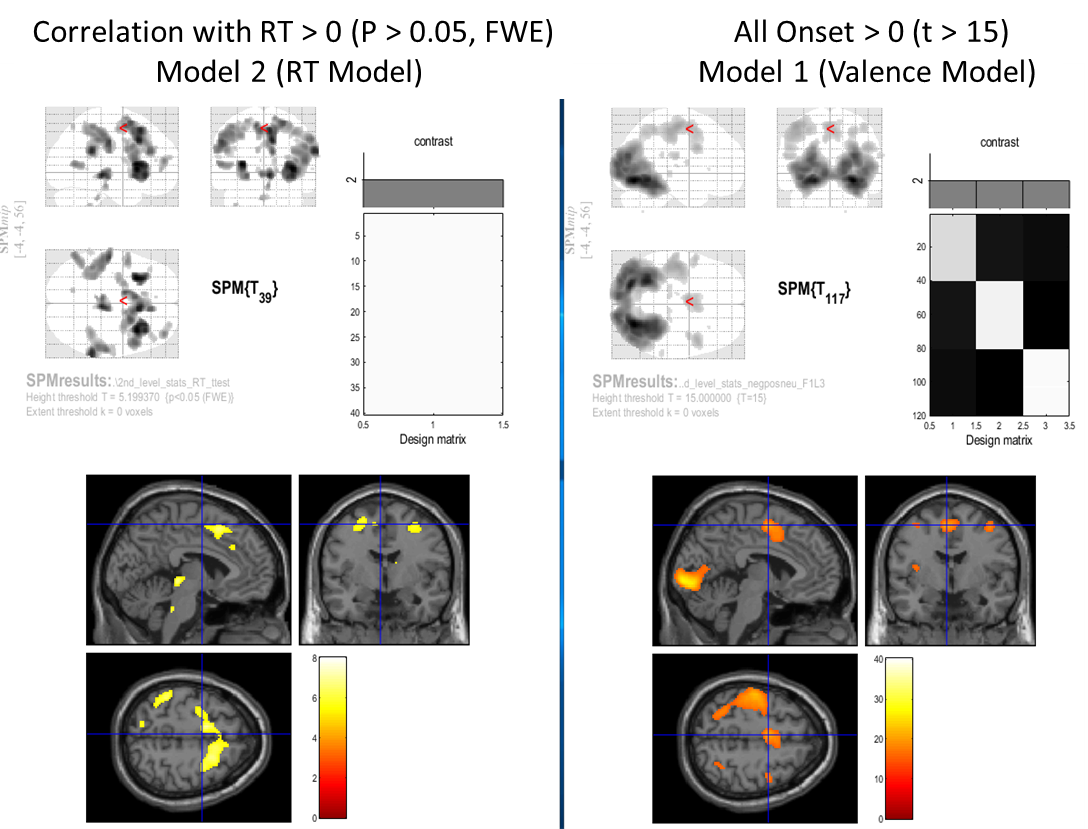


Figure S8: Comparing the parametric RT contrast from Fig. S5 (P < 0.05 FWE) with the contrast “All Onsets > Baseline” from Model 1 (Valence Model) (T-value > 15 for illustrative purposes) centering medially on the supplementary motor area.


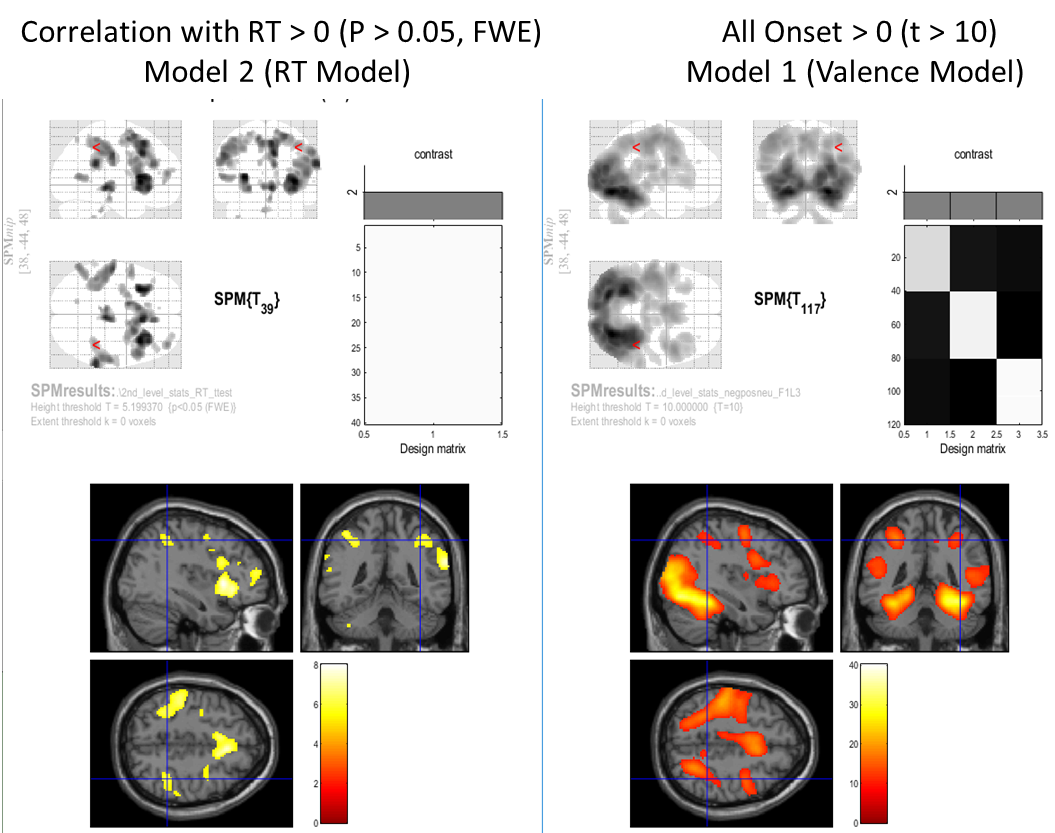


Figure S9: Comparing the parametric RT contrast from Fig. S5 (P < 0.05 FWE) with the contrast “All Onsets > Baseline” from Model 1 (Valence Model) (T-value > 10 for illustrative purposes) centering on the right parietal cortex.


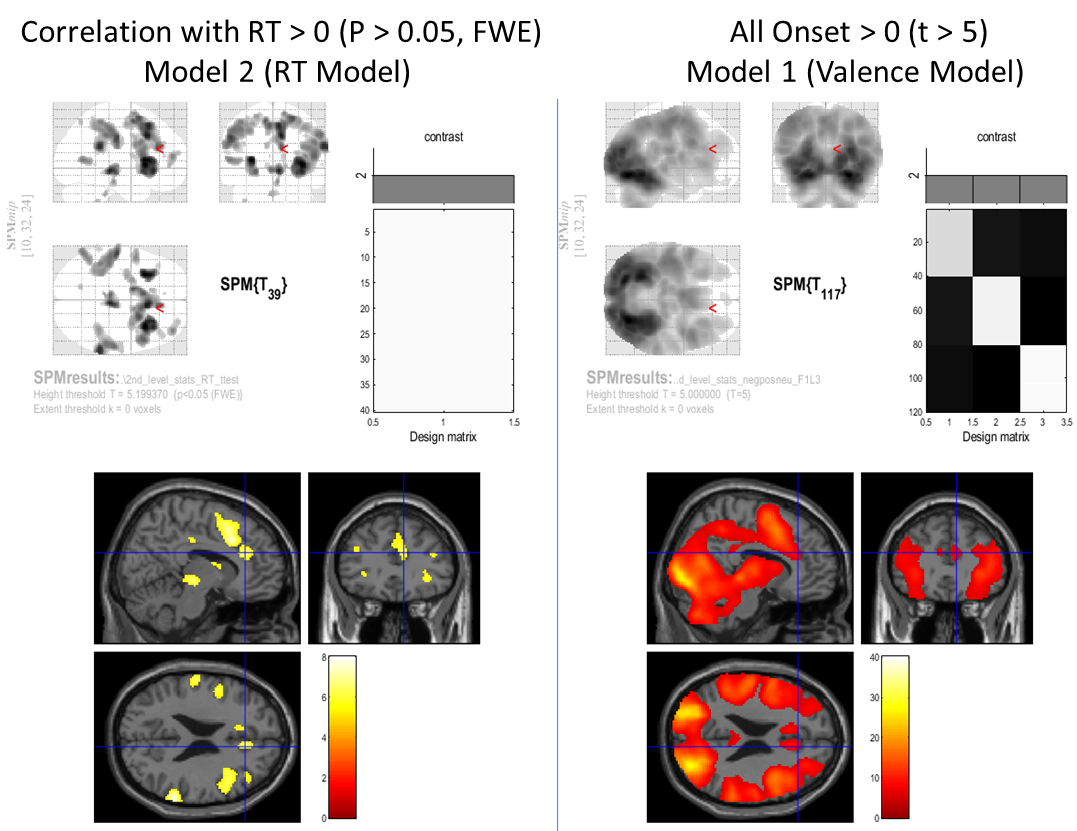


Figure S10: Comparing the parametric RT contrast from Fig. S5 (P < 0.05 FWE) with the contrast “All Onsets > Baseline” from Model 1 (Valence Model) (T-value > 5 for illustrative purposes) centering medially on the dorsal anterior cingulate cortex (dACC).


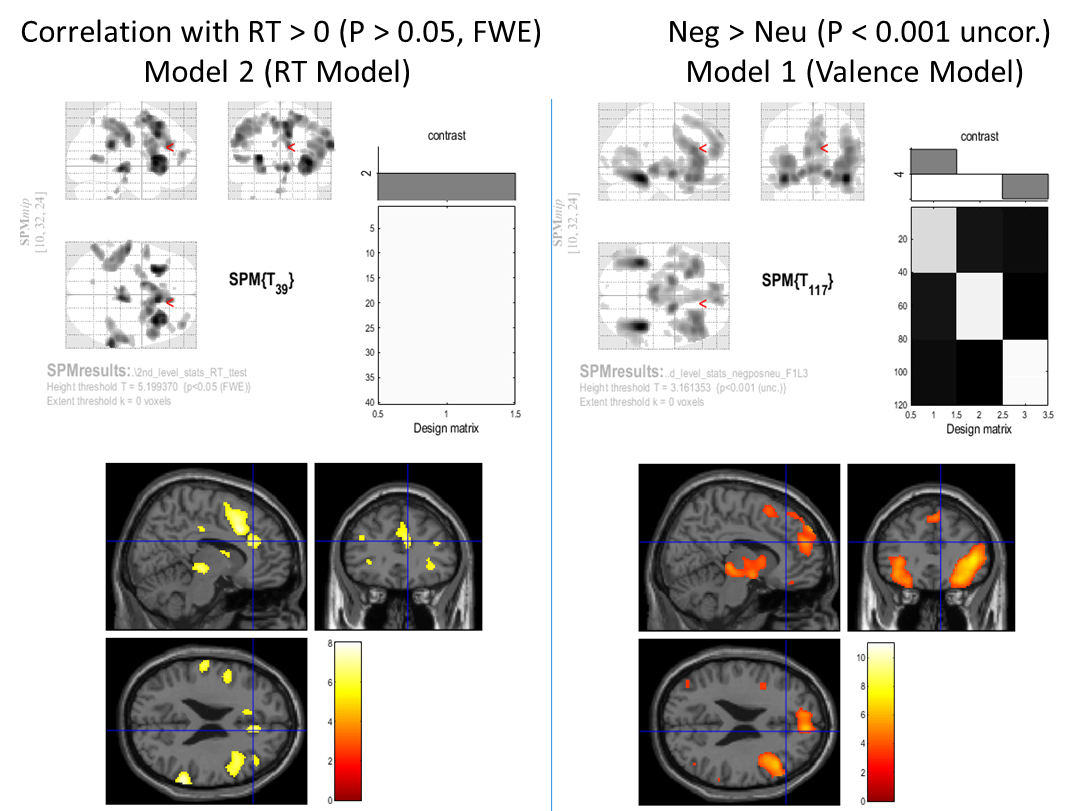


Figure S11: Comparing the parametric RT contrast from Fig. S5 (P < 0.05 FWE) with the contrast “Negative Distractors > Neutral Distractors” from Model 1 (Valence Model) (P < 0.001 uncor.) centering medially on the dorsal anterior cingulate cortex (dACC).


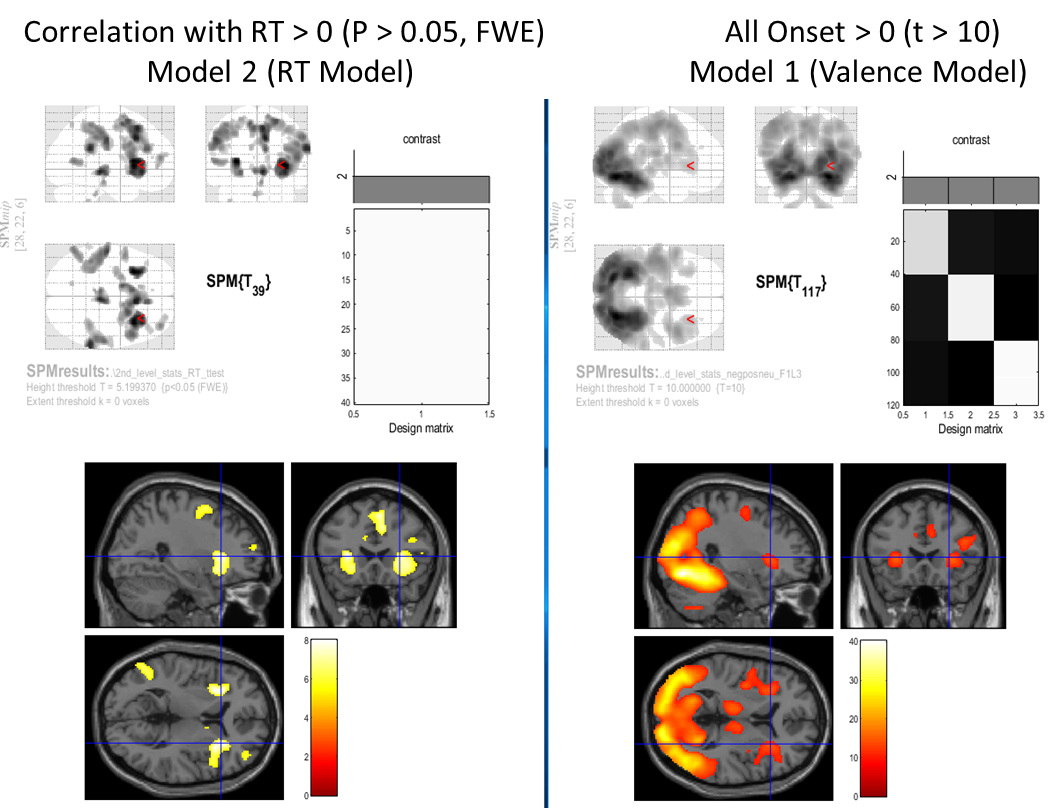


Figure S12: Comparing the parametric RT contrast from Fig. S5 (P < 0.05 FWE) with the contrast “All Onsets > Baseline” from Model 1 (Valence Model) (T-value > 10 for illustrative purposes) centering on the anterior insula.

Table S4: Details on the images used within the ACES task. Images not part of the IAPS (5-digit image numbers) were rated in-house by 20 men and 21 women. The same set of images was used for males and females but the valence category may be different for each gender.
